# Surgical Management of Chronic Pancreatitis: A Systemic Review

**DOI:** 10.7759/cureus.35806

**Published:** 2023-03-06

**Authors:** Hafiz Bilal Zafar

**Affiliations:** 1 Department of Hepatobiliary and Liver Transplant Surgery, Hamad Medical Corporation, Doha, QAT

**Keywords:** pancreas, surgery, pancreaticojejunostomy, pancreaticoduodenectomy, pancreatitis

## Abstract

Chronic pancreatitis is a debilitating disease. It is caused by the progressive destruction of normal pancreatic parenchyma, which is replaced by fibrous tissue causing pain in addition to pancreatic insufficiency. There is no single mechanism of pain in chronic pancreatitis. Several medical, endoscopic, and surgical treatment strategies are available to control this disease. Surgical techniques are divided into resection, drainage, and hybrid procedures. The review aimed to compare various surgical procedures used in the management of chronic pancreatitis. The ideal operation is the one that effectively and persistently relieves the pain and has the least morbidity with favorable pancreatic reserve. All the randomized control trials from inception to January 2023, which fulfilled the inclusion criteria, were extensively searched on PubMed and a systemic review was conducted comparing the surgical outcomes of the variety of operations used in chronic pancreatitis. Duodenum-preserving pancreatic head resection is the common procedure done with favorable outcomes.

## Introduction and background

In patients known to have chronic pancreatitis (CP), relapsing upper abdominal pain is the main reason to seek surgical treatment [1,2]. A step-up approach is done, and surgery is deemed necessary when medical and endotherapy fail to relieve the pain. Almost half of the patients have surgical intervention during the disease [3]. Surgery first in cases of advanced CP has shown to be superior to endotherapy in terms of pain control and a lesser number of procedures [4]. In addition, patients having local complications due to fibrosis leading to duodenal stenosis, biliary strictures, and splenic vein thrombosis leading to gastric varices benefit from surgery. Surgery aims to relieve pain, treat complications, preserve pancreatic reserve, and improve quality of life [5].

There are several proposed mechanisms to explain the pain related to CP. It is mainly attributed to pancreatic duct hypertension, raised intrapancreatic pressure leading to pancreatic ischemia, and ultimately replacing pancreatic parenchyma with fibrotic tissue [6]. Pain relief after decompressing a dilated pancreatic duct supports the hypothesis of the origin of pain due to ductal hypertension [7]. Similarly, supplementation by pancreatic enzymes decreases intrapancreatic pressure by reducing the pancreatic exocrine stimulation and has resulted in fewer pain scores in some patients with CP [8]. Pancreatic hypertension can lead to compartment syndrome-like features resulting in ischemia, which leads to pain [9]. Surgical drainage releases this compartment effect causing relief in pain which is not achieved with endoscopic pancreatic stent placement [6]. Bile duct and duodenal stenosis are caused by repetitive fibrosis in the plane between the pancreatic head and duodenum which is termed groove pancreatitis, which compresses the neurons located in this groove causing pain [1]. Pancreatic fibrosis causes scarring of pancreatic tissue that can raise intraductal pressure causing pain, however, no direct relationship between the degree of fibrosis and pain has been established [10,11].

Classically pancreatic head has been deemed the source of pancreatic pain in most cases [12]. The optimal timing of surgical intervention has several patient and disease-related factors, with better outcomes in terms of pain control and pancreatic reserve with intervention within three years of the onset of symptoms. On the contrary, prolonged duration of disease and regular narcotic use may lead to recurrent pain even after surgery that is attributed to central pain pathways sensitization [13,14].

The evolution of surgical procedures for CP started with drainage procedures with relapse of pain in one-third of the patients. This was attributed to the pacemaker theory suggesting the pancreatic head is the culprit for the pain process in CP, which supports the notion of resection and hybrid procedures [15]. Table 1 shows the various mechanisms of pain in CP along with its treatment rationale [16].

**Table 1:** Mechanism of pain in chronic pancreatitis with treatment rationale.

Mechanism of pain	Treatment rationale
Inflammation	Pancreatic enzyme replacement, painkillers
Pancreatic duct hypertension	Decompression procedures
Pancreatic fibrosis	Resection and hybrid procedures
Retroperitoneal scarring	Celiac block
Central pain pathways sensitization	Psychosocial input

There is a spectrum of surgical procedures in the context of CP including resection, drainage, and hybrid procedures as shown in Table 2 [16]. The usual preoperative parameters assessed are dilated versus non-dilated pancreatic duct and the presence or absence of inflammatory mass in the pancreas. The critical triangle for resection is bounded by the common bile duct, duct of Wirsung, and portal vein [5]. Table 3 shows the spectrum of surgical procedures used in the treatment of CP, along with its brief description and indications. Table 4 shows the preoperative parameters and the relevant procedures.

**Table 2:** Classification of operations used in chronic pancreatitis. PD: pancreaticoduodenectomy; PPPD: pylorus-preserving pancreaticoduodenectomy

Resection	Drainage	Hybrid
PD	Puestow procedure	Frey procedure
PPPD	Partington and Rochelle modification	Berne modification
Beger procedure	Izbicki procedure	Hamburg modification
Total pancreatectomy with islet autotransplantation	-	-

**Table 3:** Description and indications of the surgical procedures used in chronic pancreatitis. DPPHR: duodenum-preserving pancreatic head resection; PD: pancreaticoduodenectomy; PPPD: pylorus-preserving pancreaticoduodenectomy

Procedure	Description	Indications
PD [5]	Resection of the pancreatic head, duodenum, distal bile duct, and antrum of the stomach	Focal disease at pancreatic head. Bile duct stricture. Duodenal stenosis. Suspicious pancreatic head mass.
PPPD [17]	Resection of the pancreatic head, duodenum, and distal bile duct	Same as PD
DPPHR [18]	Resection of the pancreatic head and distal bile duct. It has several variations including Beger, Frey, Berne, and Hamburg	Pancreatic head mass with low suspicion of cancer
Beger procedure [19]	Resection of the pancreatic head at the portal vein, while sparing the duodenum with a rim of pancreatic tissue followed by a dual pancreaticojejunostomy. The bile duct is preserved	Same as DPPHR
Frey procedure [20]	Coring out of the pancreatic head away from the portal vein while preserving the duodenal blood supply and bile duct, followed by a lateral pancreaticojejunostomy	Same as DPPHR. Useful in the setting of portal hypertension with large collaterals
Berne modification [21]	The pancreatic head is incised anteriorly and excised entirely leaving a rim of tissue on the portal vein. The exposed bile and pancreatic ducts are incorporated by an interposed jejunal loop	Same as DPPHR. Useful in the setting of portal hypertension with large collaterals
Hamburg modification [21]	Wider resection of the pancreatic head and V-shaped incision of the anterior pancreas followed by a lateral pancreaticojejunostomy	Same as DPPHR. Useful in the setting of the non-dilated pancreatic duct
Izbicki procedure [22]	Longitudinal V-shaped incision on the anterior surface of the pancreas incorporating secondary and tertiary order ducts followed by a lateral pancreaticojejunostomy	No pancreatic head mass with a non-dilated pancreatic duct
Puestow procedure [23]	Transversely splitting the pancreas duct from tail to body just crossing the portal vein followed by lateral a pancreaticojejunostomy. Classic Puestow procedure includes splenectomy	No pancreatic head mass with a dilated pancreatic duct
Partington and Rochelle modification [24]	The pancreatic duct is open from the tail to just the right of the portal vein followed by a lateral pancreaticojejunostomy. The spleen and pancreatic tail are preserved	Same as the Puestow procedure
Total Pancreatectomy with islet autotransplantation [25]	It requires the facility to isolate islet cells from the resected pancreas. The total volume required is 0.25 mL/kg with temporary occlusion of the portal vein if portal pressure crosses 25 cm of H_2_O	Hereditary chronic pancreatitis with no risk of malignancy preferably in younger patients

**Table 4:** Preoperative parameters and relevant procedures. DPPHR: duodenum-preserving pancreatic head resection; PD: pancreaticoduodenectomy; PPPD: pylorus-preserving pancreaticoduodenectomy

Preoperative parameters	Procedures
Pancreatic head mass with suspicion of malignancy	PD and PPPD [5]
Pancreatic head mass with dilated pancreatic duct	DPPHR Beger procedure [19]. Frey procedure [20]. Berne modification [21]
Pancreatic head mass with small pancreatic duct	Hamburg modification [21]
Dilated pancreatic duct with no head mass	Puestow procedure [23]. Partington and Rochelle modification [24]
Small pancreatic duct with no head mass	Izbicki procedure [22]
Hereditary pancreatic syndromes, diffuse pancreatic parenchymal disease, refractory disease in young	Total pancreatectomy with islet autotransplantation [26]

This review aimed to compare various operations used in the treatment of CP in terms of surgical and clinical outcomes.

## Review

Methodology

Search on PubMed from inception to January 2023 was done with the following keywords “surgery in chronic pancreatitis.” The search was limited to human subjects with no language limitations. The references of the included articles were explored to find additional relevant studies. The inclusion criteria involved randomized control trials (RCTs) with fully available texts comparing the following procedures in the setting of CP: pancreaticoduodenectomy (PD), pylorus-preserving pancreaticoduodenectomy (PPPD), duodenum-preserving pancreatic head resection (DPPHR), the Frey procedure, the Beger procedure, and the Berne modification. The RCTs including non-surgical treatment of CP and management of complications of acute pancreatitis were excluded. The description and quality of the extracted data along with an analysis of the results and their interpretation were performed. The purpose was to combine the information from various sources to fulfill the aim of the study.

**Figure 1:**
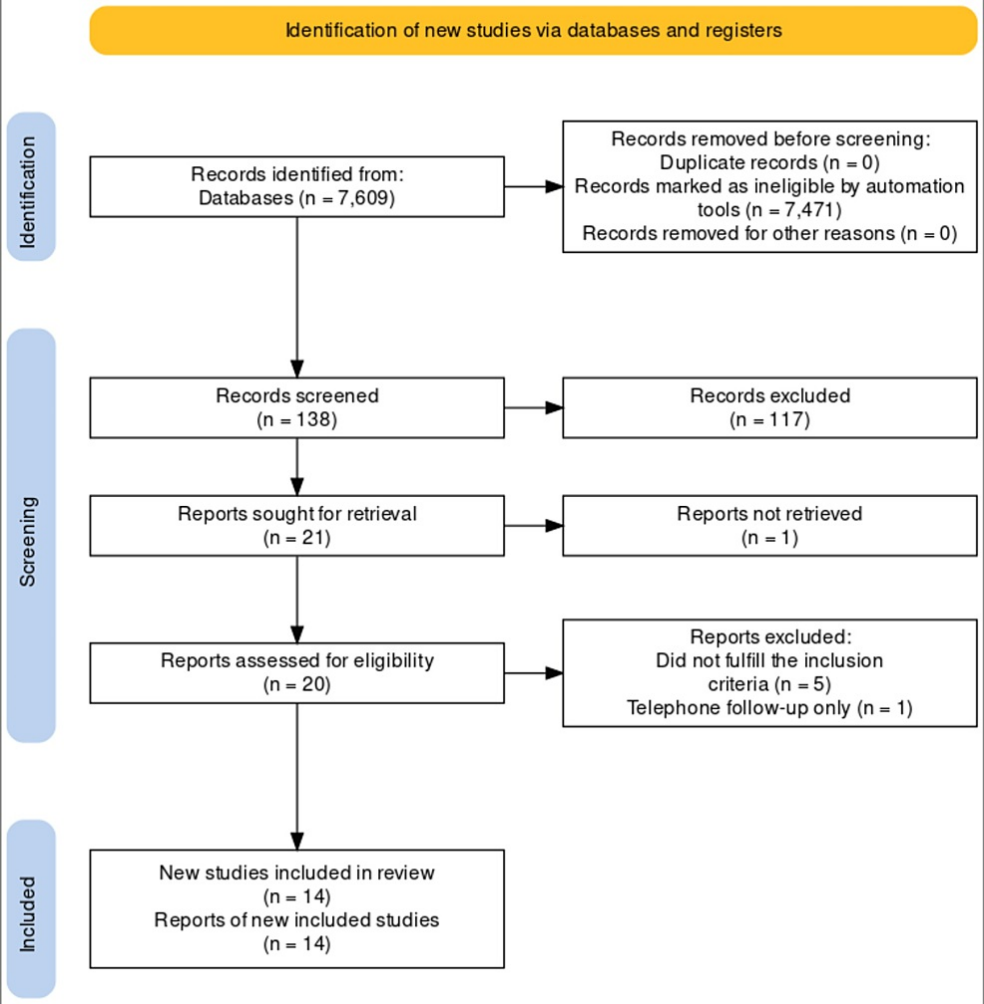
PRISMA-S flowchart describing the search strategy. PRISMA: Preferred Reporting Items for Systematic reviews and Meta-Analyses

A total of 7,609 articles were identified, of which 138 were screened out as RCTs. One hundred seventeen articles were excluded by title only. Twenty-one articles were assessed for full text. Five articles were excluded as they didn’t fulfill the inclusion criteria, and another study was excluded as it was a telephone follow-up with no concrete data. In total 14 studies were included in the review. Figure 1 shows the Preferred Reporting Items for Systematic reviews and Meta-Analyses (PRISMA)-S flowchart describing the search strategy [27].

Results

By the search strategy and inclusion criteria, 14 RCTs were included. A comprehensive analysis of the included studies was done. The surgical procedures were compared with key outcomes. Table 5 shows the included RCTs with the relevant data.

**Table 5:** Randomized control trials comparing surgical procedures for the treatment of chronic pancreatitis. DPPHR: duodenum-preserving pancreatic head resection; M: months; NA: not available; NSD: no significant difference; PD: pancreaticoduodenectomy; PPPD: pylorus-preserving pancreaticoduodenectomy; Y: year(s); QOL: quality of life

Author	Year	Country	Groups	Patient number	Morbidity	Mortality	Pain relief	New onset DM	QOL score	Follow-up
Klempa et al. [28]	1995	Germany	PD, DPPHR	21, 22	NA	0%, 5%	60%, 70%	38%, 12%	NA	5 Y
Büchler et al. [29]	1995	Germany	PPPD, DPPHR	20, 20	NA	0%, 0%	40%, 75%	NA	NA	6 M
Izbicki et al. [30]	1995	Germany	Beger, Frey	20, 22	20%, 9%	0%, 0%	95%, 94%	0%, 0%	67%, 67%	1.5 Y
Izbicki et al. [31]	1997	Germany	Beger, Frey	38, 36	32%, 22%	0%, 0%	95%, 93%	NSD	67%, 67%	5.1 Y
Izbicki et al. [32]	1998	Germany	PPPD, Frey	30, 31	53%, 19%	0%, 3%	NSD	NA	43%, 71%	2 Y
Strate et al. [33]	2005	Germany	Beger, Frey	38, 36	NA	31%, 32%	NSD	56%, 60%	66%, 58%	9 Y
Farkas et al. [34]	2006	Hungary	PPPD, DPPHR	20, 20	40%, 0%	0%, 0%	90%, 85%	15%, 0%	NA	1 Y
Müller et al. [35]	2008	Germany	Beger, PPPD	20, 20	NA	25%, 25%	NSD	46%, 78%	NSD	14 Y
Strate et al. [36]	2008	Germany	PPPD, Frey	30, 31	NA	NA	NSD	65%, 61%	NSD	7 Y
Köninger et al. [37]	2008	Germany	Berne, Beger	33, 32	21%, 20%	0%, 0%	NA	NA	71%, 66%	2 Y
Keck et al. [38]	2012	Germany	PPPD, DPPHR	43, 42	NSD	0%, 0%	67%, 67%	NSD	NSD	5 Y
Bachmann et al. [39]	2013	Germany	Frey, PPPD	32, 32	NA	30%, 53%	60%, 30%	NA	100%, 60%	15 Y
Bachmann et al. [40]	2014	Germany	Beger, Frey	38, 36	NA	39%, 34%	NSD	87%, 86%	NSD	16 Y
Diener et al. [41]	2017	Germany	DPPHR PD	125, 125	64%, 52%	7%, 3%	NSD	4%, 5%	73%, 75.3%	2 Y

PD Versus DPPHR

Klempa et al. compared PD and DPPHR with 21 patients in the PD group versus 22 patients in the DPPHR group with follow-up for five years. There was favorable pain control in both groups, however, DPPHR had significantly better outcomes in terms of pancreatic endocrine control [28]. Diener et al. compared partial PD with DPPHR with 125 patients in each group with follow of two years in multi-center settings. Partial PD included PPPD and standard PD while DPPHR included all its variations, e.g., Beger, Frey, and Berne. There was no difference in the quality of life and morbidity in both procedures [41].

PPPD Versus DPPHR

Büchler et al. compared PPPD with DPPHR with 20 patients in each group with a follow-up of six months. DPPHR showed significantly better results in terms of pain control, weight gain, and pancreatic reserve as compared to PPPD [29]. Farkas et al. compared DPPHR with PPPD with 20 patients in each group with a follow-up of one year. DPPHR was superior to PPPD in terms of operation time, morbidity, length of hospital stay, and quality of life. Both procedures were effective in pain relief [34]. Keck et al. compared PPPD with DPPHR with 43 patients in the PPPD group versus 42 patients in the DPPHR group with a follow-up of five years. Both procedures were comparable in terms of morbidity, pain control, quality of life, and pancreatic function [38].

Frey Procedure Versus PPPD

Izbicki et al. compared the Frey procedure with PPPD with 31 patients in the Frey procedure group versus 30 patients in PPPD with a follow-up of two years. The rate of in-hospital complications and quality of life were significantly less in the Frey procedure group versus the PPPD group. Both procedures were effective in pain control and control of adjacent organ complications [32]. Strate et al. compared the Frey procedure with PPPD with 31 patients in the Frey procedure group and 30 patients in the PPPD group with a follow-up of seven years. Both procedures were comparable in terms of pain control, quality of life, and pancreatic function in the long term [36]. Bachmann et al. compared the Frey procedure with PPPD with 32 patients in each group with a follow-up of 15 years. The overall survival was better in the PPPD group as compared to the Frey procedure group. The physical status of the patients was better in the Frey procedure group versus the PPPD group. Both procedures were effective and comparable in pain control [39].

Beger Procedure Versus PPPD

Müller et al. compared the Beger procedure with PPPD with 20 patients in each group with a follow-up of 14 years. Both procedures were comparable in terms of pain control, mortality, and pancreatic function in the long term. The quality of life was comparable, except for the loss of appetite was more common in the PPPD group [35].

Beger Procedure Versus Frey Procedure

Izbicki et al. compared the Beger procedure with the Frey procedure with 20 patients in the Beger procedure group versus 22 patients in the Frey group with a follow-up of 1.5 years. Both procedures were comparable in terms of pain control, quality of life, resolution of CP adjacent organ complications, and pancreatic function [30]. Similar results were obtained by the same author with 38 patients in the Beger procedure group versus 36 patients in the Frey group with a follow-up of 5.1 years [31].

Bachmann et al. compared the Beger procedure with the Frey procedure with 38 patients in the Beger procedure group versus 36 patients in the Frey procedure group with a follow-up of 16 years. Both procedures were comparable in pain control, quality of life, overall survival, and pancreatic function [40]. Similar long-term comparable results were found by Strate et al. with 38 patients in the Beger procedure group versus 36 patients in the Frey procedure group with a follow-up of nine years [33].

Berne Procedure Versus Beger Procedure

Köninger et al. compared the Berne procedure with the Beger procedure with 33 patients in the Berne procedure group and 32 patients in the Beger procedure group with a follow-up of two years. The Berne procedure showed better results in terms of operative time and length of hospital stay. The quality of life was comparable in both procedures [37].

Discussion

Recurrent upper abdominal pain and failed non-surgical treatment is the main reason to seek surgery in CP. Drainage, resection, and hybrid procedures are the three surgical options available [3]. Drainage procedures are utilized in the setting of a normal pancreatic head, with dilated pancreatic duct undergoing the Puestow procedure or its Partington and Rochelle modification and a non-dilated pancreatic duct undergoing Izbicki procedure [23,24]. Patients having suspicious pancreatic head mass undergo PD or PPPD [5]. Patients having pancreatic head mass with no or low suspicion of malignancy undergo DPPHR with one variation depending on intraoperative findings as mentioned in Table 3 [18].

Historically drainage procedures have been replaced by resection and hybrid procedures because of the high incidence of recurrent pain [5]. Currently, PD including pylorus-preserving and DPPHR is the most commonly used procedure in the setting of CP [42]. Both are highly efficacious in pain control and maintaining pancreatic endocrine function [43]. The short-term morbidity and exocrine function are better in DPPHR as compared to PD [43,44]. However, the long-term outcomes are comparable [29,34,35,38].

Within DPPHR, Beger and Frey's procedures have not shown significant short- or long-term differences from each other in terms of outcomes [30-33,40]. However, the Berne technique is simpler and quicker to perform and has less hospital stay as compared to the Beger procedure, but the long-term outcomes are equivalent [37,45]. In a recent multi-center RCT with a follow-up of two years, there was no difference seen in partial pancreatectomy that includes PPPD and standard PD versus DPPHR including its different variations like Beger, Frey, and Berne [41]. However, most of the data as evident from Table 5 support DPPHR in terms of low morbidity, a better quality of life, less pancreatic endocrine dysfunction, and equally effective pain control as compared to PD and PPPD, making DPPHR along with its variations the procedure of choice for CP. No RCT is available comparing total pancreatectomy islet autotransplantation with the rest of the procedures used in the setting of CP.

There are several limitations to this review. First, the number of RCTs is limited with a small number of patients in most of the studies. Second, the follow-up period varied between studies from six months up to 16 years. Third, relief of pain which is the main indicator of successful surgery is subjectively assessed mainly by pain scales which have their own bias. Fourth, the data was not uniform and exhaustive between different studies. Lastly, the surgical expertise offered to the patients with this wide variety of surgical procedures varies between different surgeons and centers.

## Conclusions

Chronic pancreatitis carries major morbidity because of its recurrent and long-duration nature. Pain control is the main goal in the surgical treatment of chronic pancreatitis. While each procedure has its significance and applicability, the surgical spectrum has evolved. Historically most of the patients had major resections in the form of pancreaticoduodenectomy or drainage procedures like the Puestow procedure. Now the trend is more toward hybrid procedures. The long-term outcomes of resection and hybrid procedures are comparable and excellent in terms of pain control and quality of life. Over time, duodenum preserving pancreatic head resection has turned out to be the widely used procedure in the setting of chronic pancreatitis with favorable short- and long-term outcomes and is the procedure of choice for most surgeons. The care of chronic pancreatitis is complex and should be undertaken in high-volume centers preferably in multi-disciplinary settings with experienced pancreatic surgeons, radiologists, and gastroenterologists, with ancillary support from pain specialists, psychologists, dieticians, and social workers.
